# Early imaging characteristics of 62 cases of cerebral venous sinus thrombosis

**DOI:** 10.3892/etm.2012.796

**Published:** 2012-11-02

**Authors:** HUI QU, MEILAN YANG

**Affiliations:** 1 Department of Neurology, Beijing Tiantan Hospital, Affiliated Hospital of Capital University of Medical Sciences, Beijing 100050;; 2 Third Neurology Department, The Affiliated Shenzhen Futian People’s Hospital of Guangdong Medical College, Shenzhen 518033, P.R. China

**Keywords:** thrombosis, magnetic resonance spectroscopy, magnetic resonance venography, early imaging characteristics

## Abstract

This study aimed to evaluate the early imaging characteristics of cerebral venous sinus thrombosis (CVST). A retrospective analysis was conducted of the clinical and imaging data of 62 patients with CVST diagnosed by magnetic resonance imaging (MRI) and/or digital subtraction angiography (DSA). In the 62 cases, MRI (1.5 T MRI) and magnetic resonance venography (MRV) examinations were conducted for 56 cases, and 54 of these were definitely diagnosed as CVST cases. Their MRI manifestations presented punctiform and sheet-like hemorrhagic cerebral infarction and extensive brain edema while partial cases presented cerebral ventricle dilation. In addition, MRI, MRV and DSA examinations were conducted synchronously for 2l cases. Among the 20 patients whose MRI and MRV examinations were positive, 19 cases were positive by DSA examination and the coincidence rate of the two was 95.00%. The clinical manifestations of CVST lack specificity. MRI combined with MRV examination is the preferred method of diagnosing CVST.

## Introduction

Cerebral venous sinus thrombosis (CVST) is a rare cerebrovascular disease in the clinic, and it usually affects young males. Its clinical manifestations are complex and lack specificity, its causes are perplexing (1), its incidence rate accounts for 0.5–2.0% of strokes (2) and it is easily missed or misdiagnosed by clinicians. As its mortality rate is 30–50% according to early reports (3), it is considered a rare and high-risk cerebrovascular disease. With the development of imaging technology, the early diagnosis and treatment of CVST is gradually becoming possible. According to previous studies, its mortality rate has dropped to 9.4% (4,5).

Computed tomography (CT) is widely used for the early imaging of patients with CVST. Conventional CT scanning has a low sensitivity for CVST diagnosis, which is possibly associated with anatomical variations of the venous sinus. The main direct sign of acute CVST on a conventional CT image is that a cortical venous sinus or dural sinus presents a high density (6–8), and the indirect signs are low density lesions or cerebral hemorrhage in the trans-arterial innervation zone of the brain parenchyma. In addition, enhanced CT is able to show venous sinus filling and defects and is capable of showing the classic ‘empty δ sign’.

In the various phases of CVST, magnetic resonance imaging (MRI) is generally more sensitive than CT. If the existence of thrombosis in any one venous sinus is detected by MRI examination, CVST may be diagnosed (9). The main early signs of CVST in simple MRI scans include flow shadow disappearance and signal intensity changes in the venous sinus. In the first week of incidence of venous thrombosis, the T1-weighted image presents the same signal intensity as brain tissue and the T2-weighted image presents a lower signal intensity due to the increase in deoxygenated hemoglobin content. In the second week, metahemoglobin is present in the venous thrombus and the T1- and T2-weighted images present high intensity signals. In the chronic phase, with the evolution of the thrombus and the paramagnetic products of deoxygenated hemoglobin and metahemoglobin, the gradient echo- and magnetic susceptibility-weighted images present low signals (10–13). In T2-weighted images having low intensity signals, it is extremely difficult to identify normal flow shadows, and it may be necessary to use enhanced MRI or magnetic resonance venography (MRV) to assist diagnosis. The secondary signs observable by MRI include brain swelling, edema and/or hemorrhage (6). In an MRV examination, the direct signs of CVST are high flow signal loss or fuzzy edges of a normally-developed venous sinus or irregular lower blood flow signals. The former indicates complete obstruction and the latter indicates thrombosis underfilling or recanalization thrombosis following complete obstruction of the venous sinus. The indirect signs are superficial and deep venous dilation of the brain, venous stasis and collateral circulation formation (14,15).

For CVST, digital subtraction angiography (DSA) reveals non-development of the venous sinus, development delay or slowing of the vein structure accompanied by venous dilation of the cortex, scalp or face and venous inverse flow (10). Also, DSA is able to show certain veins which are not visible by CT or MRI, particularly cortical veins and certain deep vein structures. Hypoplasia or atresia of cerebral veins or venous sinus may make it not possible for MRV or CTV to be definitely diagnosed, but the venous phase in brain angiography may be clearly shown (6).

Reasonable selection of the detection methods for identifying the early characteristics of CVST may be crucial in the early diagnosis and treatment of CVST. In the current study, a retrospective analysis of the clinical and imaging data of 62 patients with CVST diagnosed by MRI and/or DSA was conducted.

## Materials and methods

### General data

A total of 62 patients who were hospitalized at Tiantan Hospital affiliated to Capital Medical University between January 2002 and July 2007 were involved in the study. There were 26 male and 36 female cases. Their ages ranged from 15 to 60 years and the average age was 30.6±16.5 years. They were admitted in the acute or subacute phase. For disease course, 15 cases were within 1 week, 36 cases were between 1 week and 1 month and 11 cases were >1 month from onset. For possible disease causes, 12.90% (8/62) cases were due to pregnancy, 16.13% (10/62) cases were due to delivery or abortion, 4.84% (3/62) were due to oral administration of contraceptives, 12.90% (8/62) were due to cerebral facial infection and for the remaining cases, the causes were unclear. For clinical manifestations, 56 cases (90.32%) presented headache; 16 cases (25.81%) presented choked papilla, 13 cases (20.97%) presented limited neural function defect, 11 cases (17.74%) presented epileptic seizure and 5 cases (8.06%) presented consciousness disorder. In addition, there were 50 cases with lumbar puncture manometry >180 mm H_2_O (1 mm H_2_O = 0.0098 kPa), accounting for 80.65% of the patients. This study was conducted in accordance with the declaration of Helsinki. This study was conducted with approval from the Ethics Committee of Capital Medical University. Written informed consent was obtained from all participants.

### Imaging examination

i) CT scanning: a Prospeed spiral CT systemic scanner (General Electric Company, Fairfield, CT, USA) was used to conduct 122 neurocranial CT examinations for 62 patients successively. Of these, 32 cases received one CT examination, 30 cases received two CT examinations and 10 cases received three CT examinationsat the Beijing Taintan Hospital. In the week after onset, 46 cases received the first CT examination. Between 1 week and 1 month after onset, 16 cases received the first CT examination. The second and third examinations were conducted up to 1.5 years after onset. ii) MRI examination: a Model 1.5-T superconducting machine (GE Company) was used to conduct enhanced scanning with conventional T1 and T2 weighted sequences and intravenous injection of a contrast agent. A total of 56 cases received MRI and MRV examinations. iii) DSA examination: a Model DSA-2000A machine (Toshiba Corporation, Tokyo, Japan) was used to conduct aortic arch angiography and cerebral angiography. A total of 32 cases received DSA examination. In this group, MRI, MRV and DSA examinations were synchronously conducted for 21 cases.

## Results

### CT examination

For the 62 cases receiving the first CT examination, 13 cases presented the direct signs of CVST and the positive rate was 20.97%, while 15 cases presented indirect signs and the positive rate was 24.19%. Among the 46 cases receiving the first CT examination within 1 week after onset, 22 cases presented direct and/or indirect signs of CVST and the positive rate was 47.83%. Among the 16 cases receiving the first CT examination within 1 week to 1 month after onset, 6 cases presented direct and/or indirect signs of CVST and the positive rate was 37.50% (Fig. l).

### MRI examination

**Figure 2:**
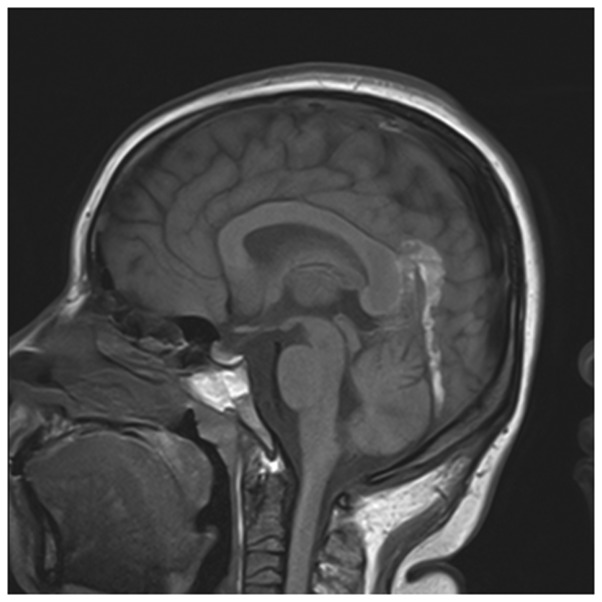
Magnetic resonance imaging (MRI) T1-weighted image displayed mixed signals for the straight sinus and vein of Galen and corpus callosum splenium swelling.

**Figure 3:**
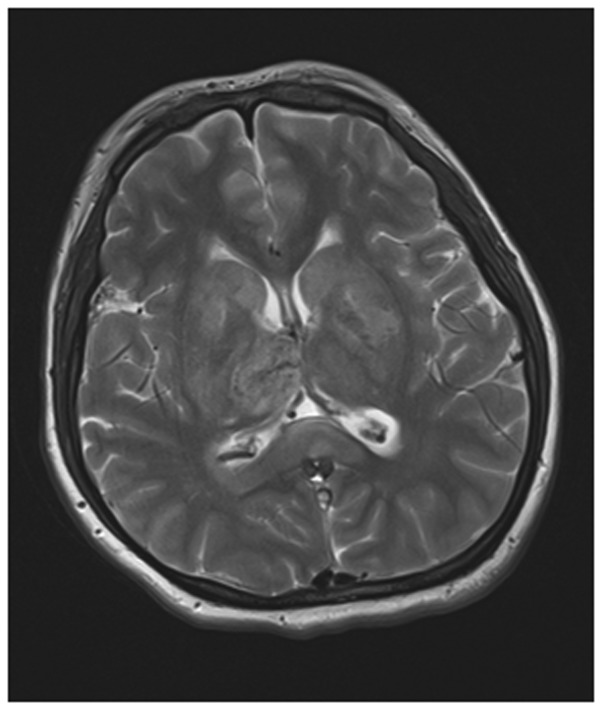
Magnetic resonance imaging (MRI) T2-weighted image displayed vein of Galen and basal ganglia with low signal intensities and corpus callosum splenium swelling.

Among the 56 cases receiving both MRI and MRV examinations, 54 cases presented adverse development or non-development of the venous sinus at lesion sites and the positive rate was 96.43%. Their MRI manifestations presented punctiform and sheet-like hemorrhagic cerebral infarction and extensive brain edema while partial cases presented cerebral ventricle dilation. According to the staging method of Isensee *et al*(4), 30 cases were in the acute phase, 18 cases were in the subacute phase and 6 cases were in the chronic phase. According to MRI, the lesions were distributed as follows: 22 cases occurred at the superior sagittal sinus, 15 cases occurred at the lateral sinus, 13 cases occurred at the sigmoid sinus, 7 cases occurred at the straight sinus and 2 cases occurred at the internal jugular vein. Five of these cases had lesions at both the superior sagittal and lateral sinuses (Figs. 2 and 3).

### DSA examination

**Figure 4:**
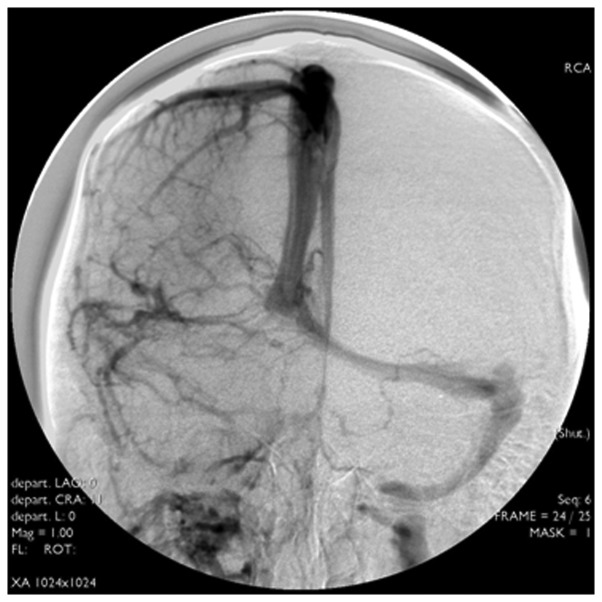
Digital subtraction angiography (DSA) showed non-development of the right lateral sinusand sigmoid sinus.

For the 32 cases receiving DSA examination, they were diagnosed with CVST. Their typical DSA manifestations were that contrast filling at the lesion sites was discontinuous, venous development was interrupted or delayed for over 5–10 sec and cortical superficial veins were dilated. Also, the backflow of partial collateral branches was established and filling was defective. With regard to the primary manifestations at lesion sites, 12 cases presented superior sagittal sinus thromboses, 3 cases presented sigmoid sinus thromboses, 4 cases presented lateral sinus thromboses and 2 cases presented straight sinus thromboses. In addition, 5 cases presented both superior sagittal sinus and lateral sinus thromboses, 4 cases presented both superior sagittal sinus and sigmoid sinus thromboses and 2 cases presented sigmoid sinus and lateral sinus thromboses (Fig. 4).

### MRI, MRV and DSA examinations

MRI, MRV and DSA examinations were synchronously conducted for 21 cases. Of the 20 cases that were positive in both MRI and MRV examinations, the DSA examination of 19 cases was also positive and the coincidence rate of the two was 95.00%. In addition, DSA examination presented as positive one case that MRI and MRV examinations presented as negative.

### Treatment and prognosis

All 62 cases received dehydration treatment. Heparin or low-molecular weight heparin treatment was administered in 38 cases, local thrombolysis with urokinase was conducted for 18 cases, mechanical thrombectomy was conducted for 4 cases and stent implantation was conducted for 2 cases. As a result, the symptoms were relieved. A total of 30 cases nearly healed, 25 cases were in the process of healing, 6 cases had left hospital and 1 case succumbed. For the healed and improved patients, no cases recurred in the follow-up period (5–12 months) and the improvement and healing rate was 88.71%.

## Discussion

The cerebral venous sinus is the main channel of cerebral venous blood backflow. CVST causes cerebral venous blood backflow disorder and induces elevation of the intracranial blood pressure to generate the corresponding clinical symptoms and signs. As the lesion sites, thromboses, elevation rates and extents of intracranial blood pressure and mechanical tolerances of the patients differ, the clinical manifestations are complex and diverse. Furthermore, CVST induces brain edema, congestion and hemorrhagic cerebral infarction in the drainage area. The lesions appear early in the course of the disease, the lesion range is wide and the lesions are not confined to the arterial innervation area. Therefore, the joint action of these effects results in the very complex and diverse clinical manifestations of CVST and severe symptoms and may even cause coma and mortality.

CT is the most popular and common craniocerebral examination technique. In CT, the direct signs of CVST include the ‘band sign’, the ‘empty δ sign’ and intravenous high density shadows, indicating thrombosis (16). The indirect signs include hemorrhagic cerebral infarction, extensive brain edema and irregular perimeters. In the early stages, the ventricle may reduce due to edema. In the advanced stages, interstitial fluid is drained into the ventricle due to an osmotic concentration increase in the ventricular wall caused by a dilated and tortuous drainage vein, which causes the ventricular enlargement. Among the patients in this group, the positive rate of CT direct signs was 20.97% and the positive rate of indirect signs was 24.19%, which was in line with the results of Renowden (14). Although the direct and indirect signs of CT have significant diagnostic values, the rate of positives is low; the band sign is observed in only 20–30% of cases and the empty δ sign in 16–46% (17). Therefore, we consider that a negative CT examination negative cannot exclude a diagnosis of CVST. For suspected cases, it is necessary to conduct MRI or DSA examination for further confirmation.

MRI is able to better reflect the pathophysiological evolution process of CVST. In addition, MRV may better reflect the blood flow state of the venous sinus, which is not influenced by thrombus signal time change, and more clearly reveal local edema and hemorrhage of the brain parenchyma (18,19). Therefore, the combination of MRI and MRV is able to provide a CVST diagnosis sensitivity reaching 90% or more (20). For the cases in the current study, the positive rate of a combination of MRI and MRV examinations was 96.43% and the coincidence rate of MRI combined with MRV and DSA examinations was 95.00%, indicating that MRI combined with MRV examination may be very useful in the early diagnosis of CVST. Considering that MRI combined with MRV examination is simple, accurate, noninvasive and reproducible and more directly and objectively reflects the thrombus site and state of blood flow and enables dynamic observation of the thrombus evolution process by multiple-angle and multiple-sequence imaging, we consider that a combination of MRI and MRV examination is the preferred method of diagnosing CVST. However, MRV has also some shortcomings. For smaller thrombi, images are unclear and signals are easily missed during imaging to cause false positives. Therefore, it is necessary to conduct DSA examination for a definite diagnosis in cases of venous dysplasia.

DSA makes it possible to judge whether there is a blood backflow disorder by dynamic observation of the cycle time of cerebral blood flow in the arterial, parenchymal, venous and venous sinus phase and thus diagnoses cerebral venous sinus disease. At present, DSA is regarded as the gold standard for diagnosing CVST (21). DSA more clearly reveals CVST, stenosis and other lesions, which is useful when conducting contact thrombolysis and mechanical thrombectomy in the venous sinus, stent implantation in the treatment of venous sinus stenosis and other interventional and disease condition monitoring treatments. However, DSA also has its limitations. For example, it does not show the thrombus itself and it has a traumatic effect and involves a certain amount of radiation. Also, certain individuals are allergic to the iodine agent and it has the risk of complications during surgery. In addition, it requires a higher technical competency and may only be conducted in a qualified hospital.

In summary, as CT, MRI combined with MRV and DSA examinations have their respective advantages and disadvantages and MRI has an excellent correspondence with DSA with regard to positive detection rate and focus distribution, our approach will be to firstly conduct CT screening for highly suspected cases according to the disease history in clinical work and then adopt the corresponding examination strategies early according to comprehensive considerations, including the disease condition of the patient, relevant hospital conditions, willingness of the patient and treatment measures. We consider that MRI combined with MRV examination is the preferred means of diagnosing CVST, while DSA examination may reduce missed diagnoses and the misdiagnosis rate. For cases requiring interventional treatment and hospitals with interventional treatment qualification, DSA may act as the preferred examination means.

## Figures and Tables

**Figure 1:**
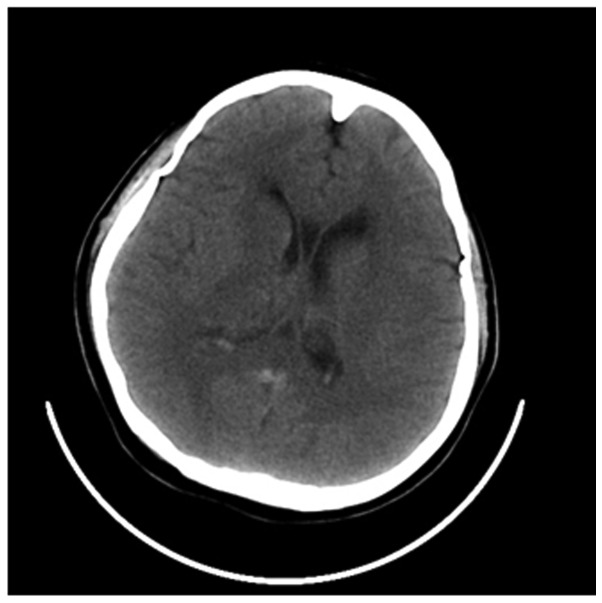
Computed tomography (CT) showed a high density vein of Galen and right thalamus swelling with decreased density.
